# Elements of Pathology

**Published:** 1838

**Authors:** 


ART. III.—ELEMENTS OF PATHOLOGY.

Dr. Drake is engaged in preparing for the press a first book of Pathology; designed, especially, for students; and to be comprised in about 450 octavo pages—price three dollars. The views embraced in it, will be, substantially, those which he has been accustomed to present, in the introductory part of his course, of public lectures, since the year 1825. Their character is, therefore, well known to many of those who are now practitioners in the Valley of the Mississippi; and they can judge, how far his contemplated work will aid them in the difficult task of instructing their students in the rudiments of pathology. He does not anticipate for it, a favorable reception East of the mountains; but is not without hope, that it will attract some share of attention in the West, and South; on which he proposes to rely, for the sale of a limited, *experimental* edition. Unable as yet to find a publisher, he expects to be compelled, either to abandon the undertaking, or publish it himself. The latter alternative will be adopted, should he find that those for whom the work is particularly intended, are desirous of procuring it. To ascertain this, he, respectfully, solicits the patrons of the Western Journal, to collect the names of such of their medical friends as may wish to subscribe for it, and forward them, through the agency of postmasters, or otherwise, free of expense, at as early a period as may be convenient. The book shall be delivered in any town, from the Lakes to the Gulph of Mexico, in which there are three subscribers. It would be desirable, to receive returns on this subject, by the first of June; when the work will be put to press, if its publication should not be given up, from the want of patronage. Should it be prosecuted, he hopes to have it ready for delivery by the first of November; that it may be made the text book of his lectures on General Pathology, to the students of the Cincinnati College, at its next session. D.

